# X-ray Computed Tomography (CT) to Scan the Structure and Characterize the Mud Cake Incorporated with Various Magnetic NPs Concentration: An Application to Evaluate the Wellbore Stability and Formation Damage

**DOI:** 10.3390/nano13121843

**Published:** 2023-06-12

**Authors:** Rasan Sarbast Faisal, Namam M. Salih, Ibtisam Kamal, Alain Préat

**Affiliations:** 1 Department of Petroleum Engineering, Faculty of Engineering, Soran University, Soran 44008, Kurdistan Region, Iraq; rsf760h@pete.soran.edu.iq; 2 Department of Chemical Engineering, Faculty of Engineering, Soran University, Soran 44008, Kurdistan Region, Iraq; 3 Research Group, Biogeochemistry & Modelling of the Earth System, Université Libre de Bruxelles, 1050 Brussels, Belgium; alain.preat@ulb.be

**Keywords:** drilling fluids, magnetite nano particles, filtration volume, mud filter cake, X-ray CT scan, wellbore stability, formation damage

## Abstract

The X-ray computed tomography method has provided unrivalled data about the characterization and evolution of the internal/external structure of materials by analyzing CTN and non-destructive imaging approach. Applying this method on the appropriate drilling-fluid ingredients plays a significant role in generating proper mud cake quality to stabilize wellbore, and avoid formation damage and filtration loss by preventing drilling fluid invasion into the formation. In this study, smart-water drilling mud containing different concentrations of magnetite nanoparticles (MNPs) was used to assess the filtration loss properties and formation impairment. Conventional static filter press, non-destructive X-ray computed tomography (CT) scan images and high-resolution quantitative measurement of CT number method were used to estimate the filtrate volume and characterize the filter cake layers, hence evaluating the reservoir damage through hundreds of merged images. The CT scan data were combined with the HIPAX and Radiant viewer digital image processing. The variation in CT number of mud cake samples under different concentrations of MNPs and without MNPs concentration were analyzed, and hundreds of 3D images as a cross-sectional profile were used. This paper highlights the importance of MNPs property in terms of minimizing filtration volume and improving mud cake quality and thickness, and hence improving the wellbore stability. From the results, a notable reduction of filtrate drilling mud volume and mud cake thickness to 40.9% and 46.6%, respectively, were recorded for drilling fluids incorporated with 0.92 wt.% of MNPs. However, this study asserts that optimal MNPs should be implemented to guarantee the best filtration property. As confirmed from the results, increasing the MNPs concentration beyond the optimal value (up to 2 wt.%) increased the filtrate volume and mud cake thickness by 3.23 and 33.3%, respectively. CT scan profile images show two layers of mud cake produced from water-based drilling fluids possessing 0.92 wt.% MNPs. The latter concentration was found to be the optimal additive of MNPs as it caused a decrease in filtration volume, mud cake thickness, and pore spaces within the structure of the mud cake. Using the optimum MNPs, the CT number (CTN) shows a high CTN and density material, and uniform compacted thin mud cake structure (0.75 mm). The produced thin mud cake layer reveals the precipitation or exchange of elemental/mineral composition during fluid-solid interaction. These results confirm that MNPs could help in avoiding or reducing the formation damage, driving away drilling fluid from the formation, and improving borehole stability.

## 1. Introduction

Formation damage is among the challenges that face the oil and gas industry and can extensively decrease recovery from subsurface reservoirs [1]. It is a relatively frequent outcome of using improper drilling mud type during drilling reservoir formation. The entrance of drilling fluid liquid into a permeable formation is known as filtration loss. Drilling fluid is the most significant part of the drilling which is used to overcome the formation pressure; therefore, it is always greater than the formation pressure [2]. Typically, the liquid from the drilling fluid will enter the formation and invade the formation for a considerable distance [3]. The process of fluid entering in the formation is known as invasion. It is the main cause of formation damage and increase in filtration loss [4]. These issues have been known for decades as abnormal productivity reduction in most reservoirs [5], and they depends on the properties of the drilling fluid and the formation permeability. When the drilling fluid liquid invades the formation, the solids from the fluid will condensate on the inner wall of the well creating a layer “Drilling Mud cake”. The filtrate liquid drives the formation fluid away from the well [6]. This particular area which is invaded by drilling fluid liquid is known as flushed zone, and the zone/s which have not been invaded by the drilling liquid is known as uninvaded zone [3]. Deep invasion effects on production due to the damages may cause a serious problem to the reservoir, as well as to the loss of fluid circulation [7]. Before the drilling operation, several tests are performed to establish the drilling mud properties, mainly mud cake and filtrate volume. Mud cake is preferred to be built up on the wall of the well while drilling. The main reason behind the significant use of mud cake is to isolate the drilling fluid from the formation [8]. Hence, selection of an appropriate drilling mud that has the ability to mitigate the problems, produce a thin compacted, robust, and impermeable filter mud cake, and reduce fluid loss into formations is the main aspect for a successful drilling program.

In connection, drilling fluid additives play a fundamental role in enhancing the drilling fluid properties. Nowadays, the drilling sector has attracted much consideration on applications of nanoparticles (NPs) with sizes less than 100 nm, due to their unique optical, magnetical, and electrical features [9]. Studies have proven that adding nanomaterials to the water-based drilling mud (WBM) has effective result due to their unique characteristics (Table 1), among them large surface area and nano-sized particles [10]. Most of the scholars have confirmed that only a small concentration of NPs is needed to plug the natural fractures, nano-pore, and micro-pore throats to guarantee a good filtration property during drilling operation [11]. The most frequent nanoparticles that are used in controlling the formation damage are ZnO, SiO_2_, Fe_3_O_4_, MgO, TiO_2_, Fe_2_O_3_, CuO, Al_2_O_3_, and CNTs [12]. NPs are used to create a thin compacted mud cake during water-based mud and reduce the damage around the wellbore vicinity [13]. During ultra-low permeable formations, such as shale, NPs successfully plug the pore throats and prevent water from penetrating into the shale formation. From the literature, mixing NPs with the drilling mud reduces fluid loss by more than 70%. This indicates that NPs are very effective in minimizing formation problems through controlling fluid loss, wettability change, slugs, and emulsion formation. Advantageously, metal oxide NPs are suitable for many conditions. Current applications of metal oxide NPs in drilling fluids delivered astonishing outcome for fluid loss control, mud cake thickness, and filtration properties [14,15,16]. 

Static filter press is a traditional method that is used to determine the mud cake thickness of any mud sample [18]. In addition to the filter press method, the role of laser apparatus and digital dial gauge method in measuring the degree of the filter cake thickness have been used by [19]. Laser method, a non-contact method of filter cake measurement, has been used to provide drilling mud cake thickness. The latter method used several circular black spots to measure the mud cake thickness of nearly 2 mm diameter and mark on a filter paper. Later, the spotted filter paper is wetted and placed on top of the mud cake. To calculate the distance of the spots from the laser head, the laser beam is applied vertically on the spots and the output signals showed by a multi-meter were measured. Data readings are taken by rotating the mud cake slowly to the up, down, left, and right, to obtain a wide range of values and hence calculate the average value [20]. Dial gauge electronic method is another technique also used to determine the mud thickness at different spots on mud cake [21]. This method is composed of a truncated cone shaped disc, transducer, and a digital display unit. The dial gauge has a tension spring which produces a weight of 25 g, the weight may damage the contact surface of mud cakes particularly for soft cakes.

Nevertheless, there are numerous uncommon methods have been described in the literature but are rarely used, such as a flat foot penetrometer, CAT scan, and ultra-sonic [20]. A number of researchers [14,22,23] studied the morphology, structure, and composition of the produced filter cake with various advanced tools (SEM, EDS, XRF, and XRD). Although these tools could give satisfying data for characterizing the mud cake, they cannot guarantee the precise characteristics and evaluation of mud cake property, quality, and layer identification compared to the data obtained from computed tomography CT scan. 

With the development of technology, more advanced scientific and complex technological tools have been applied to the study of the filter cake properties. Recently, X-ray CT scan method was used for measuring mud cake thickness. The filter cake thickness properties of water-based fluid with the CT scan method were measured by [24]. They reported that CT scan is a good approach for filter cake estimation. Their results showed that the filter cake is composed of two layers; each layer has its own property and thickness. The present study aims to:

▪ Investigate the influence of using different MNPs concentration on the filter cake quality.
▪ Examine the filter cake properties with X-ray CT scan images.
▪ Determine the filtrate volume and mud cake thickness for each of the drilling fluid samples.
▪ Measure the density of sub-millimetric/millimetric scale on surface of mud cake to determine the CTN for each of the mud cake samples.
▪ Find the optimum concentration of the MNPs that delivers a qualitative mud cake and less formation damage.
▪ Observe the CT slices variation to better characterize the mud cake layer.

Accordingly, this paper addresses the hundreds of high-resolution measurements and data from X-ray CT scan method to examine the mud cake quality and property, in order to deeply understand the role and mechanism of water-based drilling fluids incorporated with NPs in mitigation formation damage in subsurface conditions.

## 2. Experimental Studies

### 2.1. Materials and Methods

Bentonite, barite (BaSO_4_), sodium hydroxide (NaOH), tap water, xanthan gum, and magnetic oxide nanoparticle were used to prepare smart drilling mud. The chemical reagents were purchased from Merck and Aldrich chemical company, whereas bentonite and barite were supplied in powder form by a local service company. The function and composition of these materials are illustrated in Table 2. The base fluid was prepared by using bentonite as a conventional chemical additive with tap water. Electronic balance industrial scale LT1002T model was used to weigh all the material in different units. The maximum capacity of this device is 1000 g.

Firstly, 15 g of bentonite, barite 4 wt.% and 400 mL of fresh water were used constantly during the whole experimental set-up for drilling fluid preparation. At the beginning, half of the fresh water was poured into a mixing cup and 0.29 g of NaOH was added until it dissolved. The digital high shear speed mixer dispersator 220 V ± 5% AC; 50 Hz (model GJ-S5) was used to mix the solutions. Then, magnetic NPs were added. The magnetite nanoparticles (MNPs) are prepared and characterized [25]. In the previous study, the average magnetic NPs was found to be 22.99 nm. The remaining water was added and stirred with the handled mixer for 5 min to ensure fluid homogeneity. The solution was sealed in plastic containers and allowed to stand for a minimum of 16 h at room temperature, for the bentonite to hydrate. The samples were re-mixed 5 min before conducting the filtration experiments.

In addition to the static filter press method, the mud cake thickness was investigated and analyzed through the CT scan method after the filtration test. The CT-scanning test was completed on the Siemens somatom sensation 64 slice CT model, 0.24 mm to 0.33 mm isotropic resolution with fast, reliable, and outstanding quality.

### 2.2. Drilling Fluids Formulation and Testing

The base drilling fluid was prepared without the incorporation of the MNPs. Electronic balance industrial scale LT1002T model with a maximum capacity of 1000 g was used to weight the materials. Firstly, 15 g of bentonite, barite 4 wt.%, 0.29 g NaOH, and 400 mL of fresh water are used constantly as the main constituents for drilling fluid preparation. At the beginning, half of the fresh water is poured into the mixing cup and 0.29 g of NaOH is added till dissolving. The digital high shear speed mixer dispersator 220 V ± 5% AC; 50 Hz (model GJ-S5) was used to mix the solutions. Through ultrasonic mixing, the dispersion, emulsion and homogenize solution can be prepared. Then the MNPs (0.5–2 wt.%) are added. The remaining water was added and stirred with the handheld mixer for 5 min to ensure the fluid homogeneity. The solution was sealed in plastic containers and allowed to stand for 16 h at room temperature for the bentonite to hydrate. The samples were re-mixed for 5 min before conducting the filtration experiments.

In addition to the static filter press method, the mud cake thicknesses were investigated and analyzed through the CT scan method after the filtration test. The CT-scanning test was completed on the Siemens somatom sensation 64 slice CT model, 0.24 mm to 0.33 mm isotropic resolution with fast, reliable, and outstanding quality.

## 3. Results 

### 3.1. Filter Press Measurements

**Figure 1:**
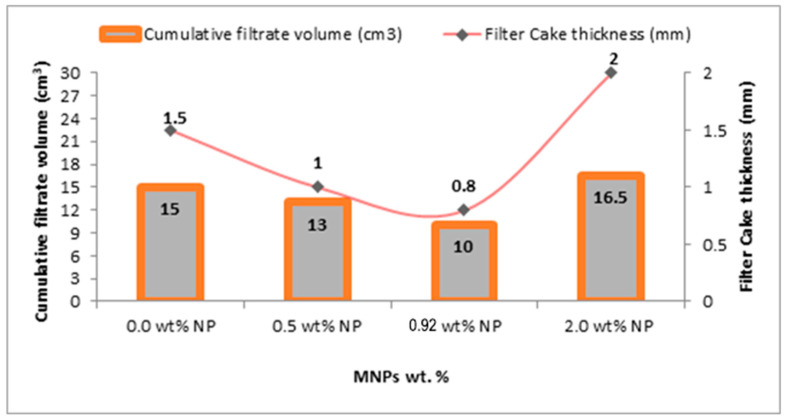
Filter cake thickness and cumulative filtrate volume for the base fluid mud and drilling mud incorporated with MNPs (0.5–2.0 wt.%).

**Figure 2:**
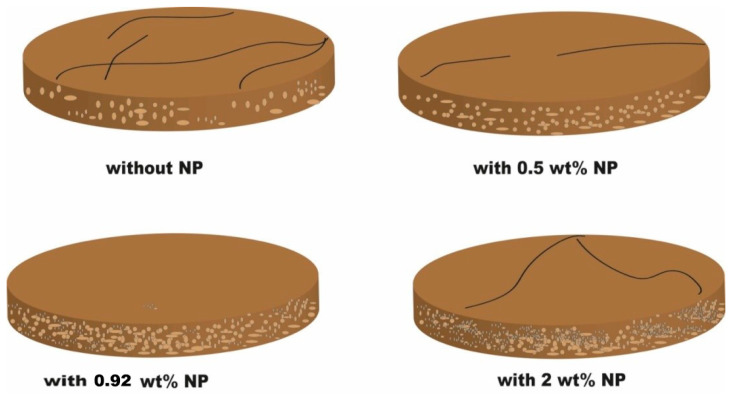
Filter cake structure from water-based drilling muds. The light brown pore spaces present the filling of these spaces by drilling mud, while the fine pores represent the agglomeration of MNPs.

The filter cake studies were conducted on smart drilling fluids possessing different concentrations of the MNPs. Table 3 illustrates the filtration characteristics of the drilling fluids that have different MNP concentrations. Figure 1 shows the filter cake thickness and cumulative filtrate volume for the drilling fluids with and without the MNPs. The condition of the static filter press was below 80 °C temperature and 100 psi pressure. Figure 2 presents the mud cake structure from drilling fluid incorporated with (0–2 wt.%) MNPs.

### 3.2. Filter Cake Analysis by X-ray CT Scan Test 

During running the CT scan tests, each mud sample was scanned from top to bottom using an X-ray source passed through the filter cake sample and detected on the other side. When one angle is scanned, one-dimensional projections of X-ray attenuation is obtained. Numerical algorithms build a two-dimensional core sample cross section from all the 1-D projections and allow the user to see inside the core sample. X-ray attenuation is proportional to density. From the high-resolution images and several hundred measurements from CT number, the user can determine the tightness of the filter cake and distribution of pore spaces through the whole mud cake. This process is 100% non-destructive. Precise quantitative data were prepared to provide visualization, to characterize the mud cake properties, and to study in detail the internal structure of the mud cake. For a single mud cake sample, the scanning time lasted about 3 h. The pixel at any point in the image can be represented by a numerical value, which is referred to as the CT number. It can be expressed as

Hu = aμ + b(1)

where a and b are constants and μ is the absorption coefficient of X-ray.

**Figure 3:**
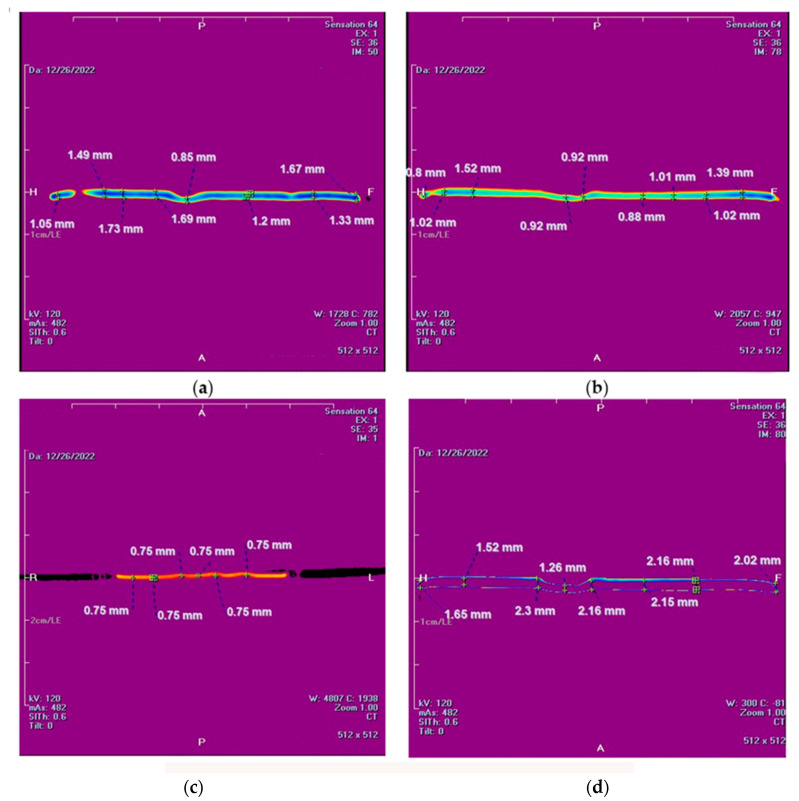
X-ray CT Scan analyses for mud cake thickness with (**a**) reference mud (**b**) 0.5% MNPs (**c**) 0.92 % MNPs (**d**) 2% MNPs.

The images were taken through the mud cake diameter (Figure 3). Unlike conventional methods, CT scan measurements are very effective for determining mud cake thickness uniformity across the mud cake diameter. Moreover, it detects cracks, pores, and other textures.

### 3.3. CT Number Analysis

**Figure 4:**
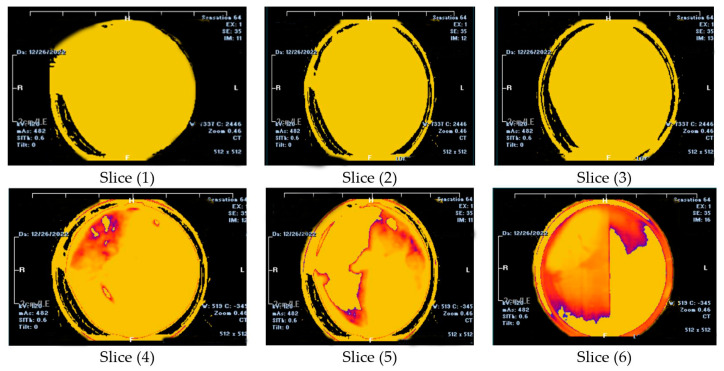
The CT Scan images for the mud cake samples containing 0.92 wt.% MNPs. The CT scan images were taken in the direction of the filtrate invasion. Slice 1 represents the top surface of the mud cake, whereas slice 6 represents the bottom surface.

**Figure 5:**
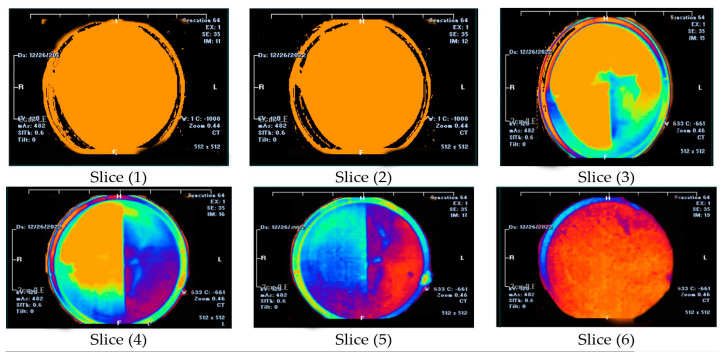
The CT scan images for the mud cake samples containing 2 wt.% MNPs. The CT scan images were taken in the direction of the filtrate invasion. Slice 1 represents the top surface of the mud cake, whereas slice 6 represents the bottom surface.

**Figure 6:**
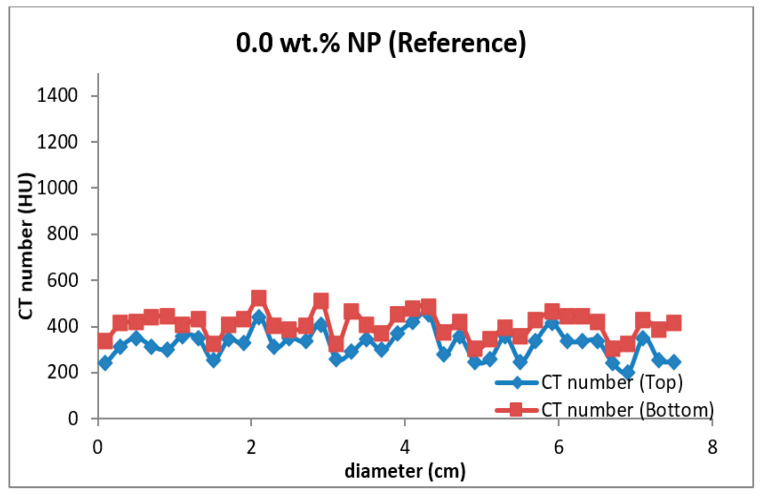
CTN through the drilling mud cake from the base drilling fluid. The red spots represented the bottom layer, and the blue spots represented the top layer.

**Figure 7:**
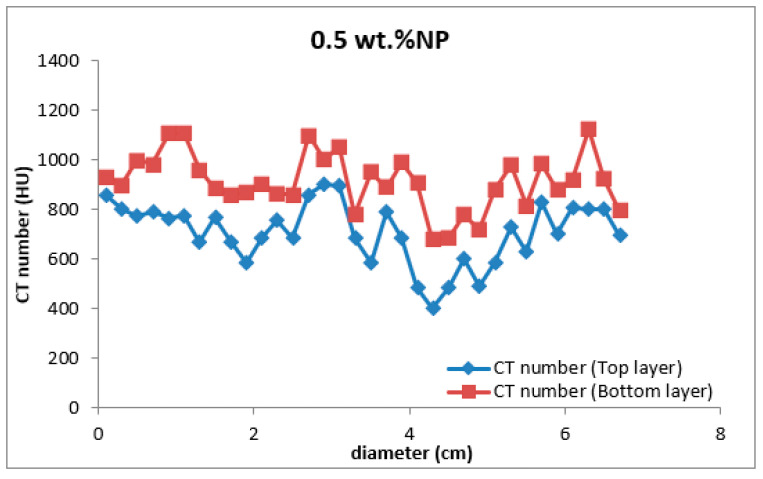
CTN through the drilling mud cake from drilling mud incorporated with 0.5 wt.% MNPs. The red spots represented the bottom layer, and the blue spots represented the top layer.

**Figure 8:**
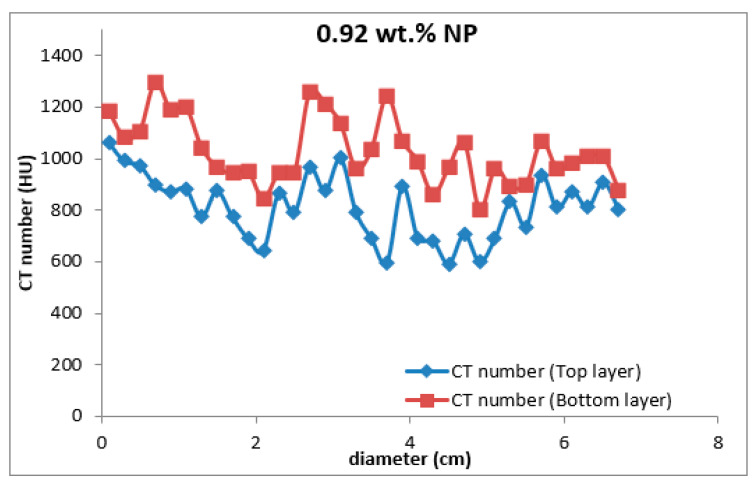
CTN through the drilling mud cake from drilling mud incorporated with 0.92 wt.% MNPs. The red spots represented the bottom layer, and the blue spots represented the top layer.

**Figure 9:**
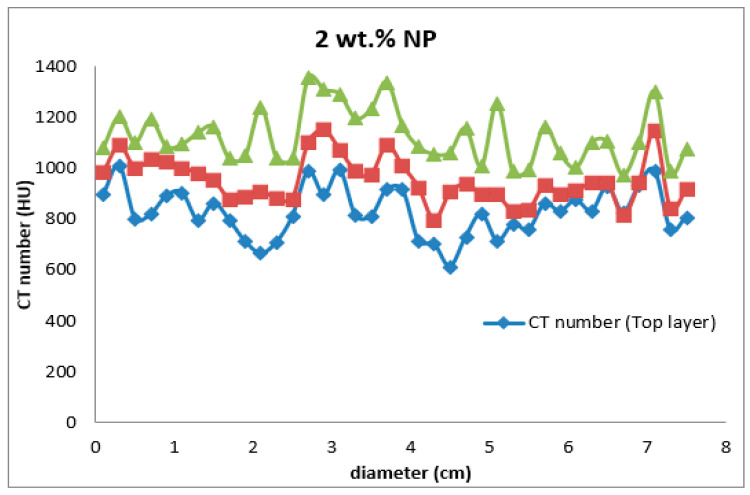
CTN through the drilling mud cake from drilling mud incorporated with 2 wt.% MNPs. The red spots represented the bottom layer, the blue spots represented the top layer, and green spots represented the third layer.

Four types of mud cake were scanned using CT scan, with different composition (base fluid without MNPs, drilling mud with 0.5, 0.92, and 2 wt.% MNPs) in direction of the filtrate invasion. The CT scan measurements of mud cake samples were analyzed by the HIPAX and Radiant viewer digital image processing software (v2.4.6), to investigate the mud cake layers (Figure 4 and Figure 5) by determining the variation of the CT numbers. Figure 6, Figure 7, Figure 8 and Figure 9 depict CT number through the mud cake diameters at different nanoparticle concentrations (0, 0.5, 0.92, and 2 wt.%). The average CT number layers for the mud cakes possessing different nanoparticle concentrations are illustrated in Table 4. These CTNs had been averaged using the complete set of data through the filter cake diameter (Figure 6, Figure 7, Figure 8 and Figure 9). The results confirmed that the CTNs for the top layer were averaged to be 321.9, 719.4, 822.9, and 827.9 HU for the filter cakes of the 0, 0.5, 0.92 and 2 wt.% MNP, respectively. The average CTNs for the bottom layer were 408.4, 921.2, 1028, and 952.8 HU for the 0.0, 0.5, 0.92 and 2 wt.%, respectively.

### 3.4. CT Scan Analysis for Filter Cake Crack and Pore Determination 

Along with the mud cake thickness, layer identification, and CT number analysis, the CT Scan test was used to detect the shape and quality of the mud cake possessing different MNPs concentrations.

## 4. Discussion

### 4.1. Static Filtration Test

The Static filter press data regarding adding MNPs to the base mud displays a significant impact on the filtration capacity. A minor increase of the MNPs concentration of 0.5 wt.% influence on the reduction of the filter cake thickness and cumulative filtrate volume by 33.3% and 30.1%, respectively, compared to the base mud. This could be due to the MNPs unique properties [9]. The best improvement for respective reduction in cake thickness and cumulative filtrate volume shows at the addition of 0.92 wt.% MNPs concentration by 46.6% and 40.9%, compared to the drilling base mud with no MNPs (Table 3 and Figure 1). However, increasing the MNPs concentration to 2.0 wt.% adversely affected the filtration properties of the drilling mud by an increase in the filter cake thickness and cumulative filtrate volume by 33.3% and 3.23%, respectively. This may result from MNPs agglomeration, which leads the MNPs to behave as larger particles, causing higher filtrate volume penetration into the formation. The results exhibit the same trend as those reported in the literature [26]. The reduction in filtration volume obtained from the tests proves the ability of the water-based drilling mud to create a thin, low-permeability mud cake layer to prevent the liquid from flowing through the filter paper. Wellbore stability is of critical importance for the drilling process since it is favorable to have an intact wellbore that is functional and fit for the purpose for which it was drilled. A damaged wellbore can impair its use, resulting in large economic losses to the operating company. Thus, wellbore instability in most of the cases, may lead to lost circulation, a stuck and damaged drill pipe and, eventually, loss of the open-hole section. Overall, drilling muds must have a low fluid loss to mitigate the problems of wellbore instability. In water-sensitive rocks such as shale formations, it is significant to formulate a drilling fluid in order to forbid formation instability issues resulting from fluid loss. From the literature, a mud cake formation with less than 1.5875 mm is considered to be an acceptable thickness [27], as thick mud cakes may result in a negative impact on subsurface reservoir formations such as a stuck pipe in highly permeable zones [28]. Thus, to produce a thin mud cake layer, the drilling mud should contain nano-sized particles to precipitate in pore spaces, in order to block the pores in permeable formations. Our results confirm that the formulation of the water-based drilling fluids composed of 0.92 wt.% of MNPs is the optimum formulation to guarantee better wellbore stability by creating thin and denser mud cake with less permeability and porosity. For deep details to evaluate the structure and composition of mud cake, a high-quality image and density measured data obtained by X-ray CT scan test are required. Our study documents for the first time an ideal mud cake structure compared to previous studies [26]. Thus, high-resolution images and HU (CTN) from X-ray CT scan would be a better alternative than conventional methods to reduce wellbore failure and at the same time to obtain a potential enhancement for drilling fluid properties.

### 4.2. X-ray CT-Scan for Evaluation of Filter Cake and Wellbore Damage 

X-ray computed tomography (CT) scan is a non-destructive imaging approach, used to detect the texture of the external and internal layers of the filter cake and core surface rocks. This technique delivers three-dimensional visualization of a wide range of materials and high-resolution quantitative data of CT numbers. This number is represented by a specific unit called Hounsfield unit (HU). Hounsfield (1979), the inventor of CT technology, defines the CT number as:

(2) $$\mathrm{HU}=\left(\frac{\mu tissue-\mu water}{\mu water-\mu air}\right)\times 1000$$

Since the absorption coefficient of air is negligibly small, therefore, Equation (2) modifies to:

(3) $$\mathrm{HU}=(\frac{\mu tissue-\mu water}{\mu water})\times 1000$$

This number reflects the degree of X-ray absorption of the tested material, and it has a linear relationship with the volume electron density of the material. Therefore, the ability of the object to absorb X-rays is related to its own density and the greater the density of the specimens, the stronger the ability to absorb X-rays [29]. The following formula represents the intensity of the X-rays.

I = I_0_ exp (−μx) = I_0_ e^−μx^ (4)

where I_0_ and I are the light intensity before and after the X-ray penetrates the specimens, respectively, with the unit of eV·m^−2^·s^−1^, x is the penetration length of the X-ray in the tested object, with the unit of cm.

X-ray CT scan is considered as a very useful tool for understanding reservoir formation damage, examining, visualizing, quantifying, and qualifying the reservoir heterogeneity. This technique is used to understand morphological and topological structures, and to identify homogeneity, heterogeneity, density, and porosity distributions of any material. Moreover, it helps to identify various zones/lithologies, fractures and allows mineralogical mapping for tight reservoirs [21]. Recently, this technique was used to characterize the mud cake thickness. Elkatatny and co-schollars [24] measured the cake thickness properties of water base fluid by CT scan method. They concluded that CT scan is a valuable potential approach for filter cake estimation. Traditional tools and models assumed that the filter cake is homogeneous whereas the CT scan detects a heterogeneity of the filter cake through observing the three-dimensional texture of the mud cake. 

The current study shows that X-ray CT scan for analyzing thickness, structure and density of filter cake, and wellbore stability allows better identification and evaluation than the conventional tools (e.g., filter press, laser, dial gauge electronic method). The mud cake thickness obtained from base fluid shows unequal thickness throughout the mud cake diameter (Figure 3a); this non-uniform mud cake structure opens the gate for the drilling fluid to press and invade the formation for a long distance, plugging the pore spaces inside the rock, and might cause a dissolution and/precipitation in the targeted spaces. Therefore, this might have negative impact on reservoir characteristics and finally could cause formation damage. Moreover, deep invasion leads to abnormal productivity reduction in the reservoir due to the formation damage. Partial or total loss of circulation mud fluids from the wellbore to the formation is another problem during drilling process. 

To get a detailed observation and evaluation about the mud cake containing non-sized particles, X-ray CT scan was utilized, adding 0.5 wt.% nanoparticle (Figure 2) to the base mud. The result displays an improvement in filter cake thickness. This improvement confirms the ability of the nanoparticle to form a thinner mud cake layer with a more uniform mud cake thickness and structure compared to the base mud. The CT numbers (CTN) from the mud cake with 0.5 and 0.92 wt.% nanoparticle produced two separate CTN populations: bottom and top layers population (Figure 7 and Figure 8), while the mud cake with 2 wt.% nanoparticle produced three layers (Figure 9). The variation of CTN along the diameter of the mud cake lines indicates the variation in density of the mud cakes (Figure 6, Figure 7, Figure 8 and Figure 9). These variations in density, especially along the top and bottom layers, indicate the significant variation in composition around these populations. The lower layer of the mud cake from drilling fluid formulation containing 0.92 wt.% MNPs reveals the prominent compacted layer and highest density with a very unique CTN, (up to 1297 HU). The high CTN from the mud cake with added nanoparticle water-based mud would add a new insight into the drilling mud. The obtained high CTN and high density mud cake have not been recorded previously; the highest from previous works was up to 560 CTN for nanoparticle water-based mud [26]. The compact and tight material from the bottom layer of the mud cake was probably caused by the saturation of MNPs into the lattice of the mud cake structure, which finally lead to the filling of the pore spaces (Figure 2). Besides, hundreds of merged images show a uniform and very thin mud cake with MNPs of 0.92 wt.%, with thickness around 0.75 mm. (Figure 3c). The uniform thin layer, evidenced by CTN as compacted mud cake structure, probably formed by precipitation or exchanging elemental/mineral composition during fluid-particle interactions [30,31]. Consequently, the dense and compacted mud cake probably prevented the formation damage, driving away drilling fluid from the formation. As a result, this improves borehole stability, and finally solves the filtration loss. 

However, our study shows that increasing magnetite nanoparticle concentration beyond the optimum (0.92 wt.%) causes an increase in the mud cake thickness with a non-uniformity of layer and wellbore instability (Figure 3d). Although the CT scan test was used in some studies for examining the mud cake layers through the CT number calculation [26], it has not been used for mud cake thickness investigation through the mud cake diameter as shown in Figure 3. Our study, for the first time, highlights the importance of CT scan measurement for mud cake thickness identification through all the mud cake diameter, and confirms the similarity of the mud cake thickness along the whole diameter or not. In other words, the CT scan test can detect mud cake thickness uniformity through the mud cake diameter. 

**Figure 10:**
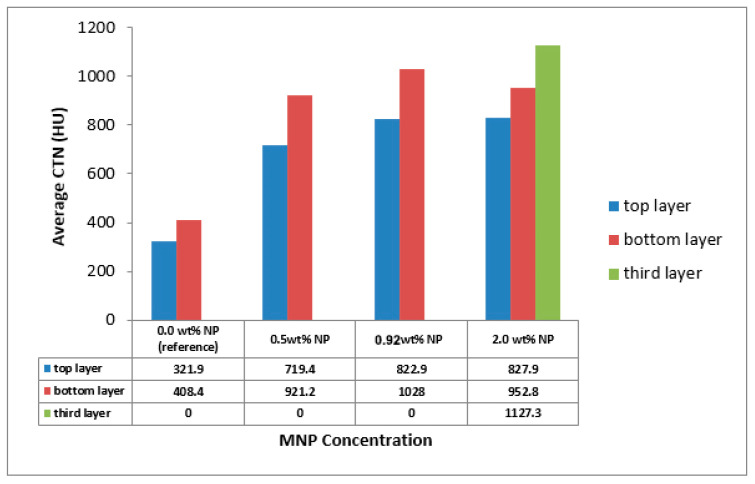
Average mud cake CTN versus the MNPs concentration.

The CT scan analyses were used to detect the mud cake layers by calculating the CT number of the prepared mud cake. The top surface of the mud cake (close to the drilling fluid) was represented by slice 1, and slice 6 represents the bottom surface (close to the formation). The variation of the image colors indicates the change in density (CTN). CT scan was carried out on a mud cake sample from drilling fluid incorporated with 0.92 wt.% MNPs, showing two layers along the prepared mud cake. Depending on the CT scan image, slices 1 to 3 represent the top layer and slices 4 to 6 represent the bottom layer (Figure 4). The mud cake sample made up from drilling fluid incorporated with 0.92 wt.% MNPs shows a similar result to [15]. On the other hand, the mud cake sample with 2 wt.% MNPs (Figure 5) consists of three layers. Slices 1 and 2 represent the top layer, slices 3 and 4 the bottom layer, and slices 5 and 6 the third layer, or nanoparticle layer, since it was formed by the nanoparticle agglomeration and settled down below the bottom layer with an average CTN of 1127.3 HU (Figure 10). A similar scenario was also observed by [26] with increased NPs concentration from 1.5 to 2.5 wt.%, a third layer showed lower average CT number of 545.37 HU. Thus, CTN confirms the stability of the bottom layer, and MNPs play an astonishing role in building the mud cake texture. The CTN of the filter cake from drilling fluid containing 2 wt.% MNPs shows a high CTN compared to filter cake from drilling fluid containing 0.92 wt.% MNPs.

The former compositions suggest a significant MNPs agglomeration and formation of a third layer, close to the formation. The three layers obtained from CT images in our study have not been reported previously, however, ref. [15] documented only a CTN of the third layer, without illustrating the 3-D visualizing images. Our results show high average CTN and more dense mud cake layers compared to those reported by [26]. The later calculated the average CTN of the top layer to be 292.89 HU, and for the bottom layer 384.78 HU for the filter cake samples made up from drilling fluids contain 0.5 wt.% ferric oxide and silica NPs. The recent CTN from the third layer could be linked to the drilling mud composition and the MNPs efficiency in creating a more compact and denser mud cake with less porous and permeable texture. Nevertheless, CTN shows the adverse impact of filter cake characteristics with increasing MNPs concentration beyond the optimum value.

**Figure 11:**
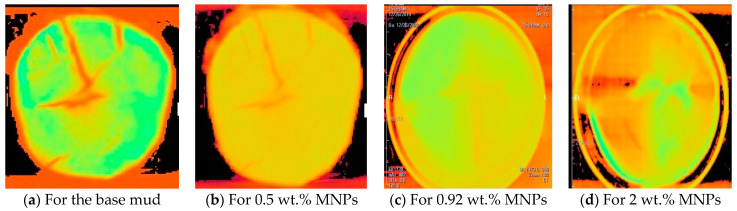
CT scan test for the mud cake with (**a**) based-drilling mud (0.0 wt.% MNP), (**b**) for 0.5 wt.% MNP, (**c**) for 0.92 wt.% MNPs, (**d**) for 2 wt.% MNPs.

Furthermore, X-ray CT scan data displays a highly permeable and porous structure for the mud cake obtained from water-based drilling mud, besides the scanned images show a highly cracked surface of the mud cake. Such a mud cake would bring a high risk for drilling operation, since highly permeable mud cake formation will allow further drilling fluid diffusion to the formation, and high porosity mud cakes will allow more drilling fluid solids to accumulate on the wall leading to thicker mud cakes [14,15]. However, with only 0.5 wt.% MNPs concentration the cracks disappeared with low pore space texture. The latter data probably influenced the mud cake quality (Figure 11b). A thin compacted, impermeable, and non-porous mud cake layer with no fractures and cracks were achieved with 0.92 wt.% MNPs concentration (Figure 11c). This indicates the strong ability of MNPs to create bonds between the particles in order to create a denser mud cake structure. Increasing MNPs concentration to 2 wt.% (above the optimum concentration) promoted a cracked surface and fractures in the mud cake, these cracks are linked to the nanoparticle agglomeration (Figure 11d). 

Consequently, the drilling fluids with 2 wt.% MNPs cannot properly plug the tiny pores due to the large and variable sizes of nanoparticles agglomeration. Thus, the optimum MNPs concentration that should be used to create a better mud cake structure is 0.92 wt.%. 

## 5. Conclusions

Water-based drilling fluids were prepared by adding a set sequence of MNPs concentrations (0, 0.5, 0.92, and 2 wt.%) to produce a various kind of mud cake samples. The main purpose of adding a set sequence of MNPs into water-based drilling fluids is to control the filtration loss, wellbore stability, and formation damage during drilling process under temperature of 80 °C with pressure of 100 psi. X-ray CT-scan was utilized to evaluate the produced mud cake, and eventually the wellbore stability, through merging hundreds of 3-D visualization images and high-resolution qualitative measurements of CT numbers. It allowed estimation of the density, structure, and quality of mud cake. The conclusions observed from this study are summarized as the following:

1. The produced mud cake from the water-based drilling fluid without adding the MNPs additives are characterized by high thickness, low density, and high pore-space texture with cracked and irregularities surface. These properties are based on the observation obtained from images contrast and CTN from X-ray CT scan.
2. Filter cakes from drilling fluid incorporated with 0.92 wt.% MNPs show optimum filtration loss performance. In addition, when the optimum MNP concentration (0.92 wt.%) is used in the drilling mud solution, the filtrate volume and mud cake thickness were reduced by 40.9% and 46.6%, respectively. Results yielded smoother filter cake morphology, low porosity, and permeability microstructure. Thus, it avoids the drilling mud particles penetration into the formation, reduces the reservoir damage, and improves the wellbore stability.
3. CTN data proved that the deposited mud cake sample from drilling fluid of optimum formulation of the MNPs consists of two layers. The layer near to the formation is the main layer (bottom layer) in which MNPs play a big role in creating a good microstructure. On the other hand, increasing MNP concentration to 2 wt.% resulted in loss of filtration control and formation of a new layer (third layer) containing the agglomerated MNPs below the second layer, which undesirably affects the well bore stability filter cake quality.
4. The utilization of X-ray CT scan for filter mud cakes is an efficient approach for understanding, examining, visualizing, quantifying and qualifying the impact of nano-additives incorporated in drilling fluids on wellbore stability and formation impairment.

## Figures and Tables

**Table 1:** Physical and chemical NPs properties [9,17].

Chemical Properties	Physical Properties
Increased chemical reactivity or stability. Improve thermal conductivity. Accelerate solubility under appropriate conditions.	A larger surface area relative to volume. Greater mechanical strength. Improve optical properties increases electrical conductivity aggregation rate increases

**Table 2:** The main ingredients of drilling fluids and their functions.

Material	Composition	Function
Tap water	400 mL	The aqueous media
Bentonite	15 g	Lubricates and cools the cutting tools while protecting against corrosion
Barite	4 wt.%	Weighting agent
Xanthan	0.1 g	Viscosity modifier
NaOH	0.29 g	Adjusting the pH range
MNPs	0.5–2 wt.%	Rheology and filtration loss modifier

**Table 3:** Filtration characteristics of the base drilling fluid and drilling fluids containing different content of MNPs (0.5–2 wt.%).

MNPs (wt.%)	Filter Cake Thickness (mm)	Percentage Change in Thickness (%)	Cumulative Filtrate Volume (cm^3^)	Percentage Change in Filtrate Volume (%)
0	1.5	Base fluid	18.6	Base fluid
0.5	1	−33.3	13	−30.1
0.92	0.8	−46.6	11	−40.9
2	2	+33.3	19.2	+3.23

**Table 4:** Average CT number layers for the mud cakes possessing different magnetite nanoparticle concentrations.

MNPs Concentration (wt.%)	Top Layer	Bottom Layer	Third Layer
0.0	321.9	408.4	-
0.5	719.4	921.2	-
0.92	822.9	1028	-
2.0	827.9	952.8	1127.3

## Data Availability

Not applicable.
